# A new diagnostic approach for the identification of patients with neurodegenerative cognitive complaints

**DOI:** 10.1371/journal.pone.0217388

**Published:** 2019-05-24

**Authors:** Sabah Al-Hameed, Mohammed Benaissa, Heidi Christensen, Bahman Mirheidari, Daniel Blackburn, Markus Reuber

**Affiliations:** 1 Dept of Electronic and Electrical Engineering, University of Sheffield, Sheffield, United Kingdom; 2 Dept of Computer Science, University of Sheffield, Sheffield, United Kingdom; 3 Centre for Assistive Technology and Connected Healthcare, University of Sheffield, Sheffield, United Kingdom; 4 Sheffield Institute for Translational Neuroscience (SITraN), University of Sheffield, Sheffield, United Kingdom; 5 Academic Neurology Unit, University of Sheffield, Royal Hallamshire Hospital, Sheffield, United Kingdom; Nathan S Kline Institute, UNITED STATES

## Abstract

Neurodegenerative diseases causing dementia are known to affect a person’s speech and language. Part of the expert assessment in memory clinics therefore routinely focuses on detecting such features. The current outpatient procedures examining patients’ verbal and interactional abilities mainly focus on verbal recall, word fluency, and comprehension. By capturing neurodegeneration-associated characteristics in a person’s voice, the incorporation of novel methods based on the automatic analysis of speech signals may give us more information about a person’s ability to interact which could contribute to the diagnostic process. In this proof-of-principle study, we demonstrate that purely acoustic features, extracted from recordings of patients’ answers to a neurologist’s questions in a specialist memory clinic can support the initial distinction between patients presenting with cognitive concerns attributable to progressive neurodegenerative disorders (ND) or Functional Memory Disorder (FMD, i.e., subjective memory concerns unassociated with objective cognitive deficits or a risk of progression). The study involved 15 FMD and 15 ND patients where a total of 51 acoustic features were extracted from the recordings. Feature selection was used to identify the most discriminating features which were then used to train five different machine learning classifiers to differentiate between the FMD/ND classes, achieving a mean classification accuracy of 96.2%. The discriminative power of purely acoustic approaches could be integrated into diagnostic pathways for patients presenting with memory concerns and are computationally less demanding than methods focusing on linguistic elements of speech and language that require automatic speech recognition and understanding.

## Introduction

Memory complaints are common, increase with age and are a major reason for primary care consultations. There is an increasing emphasis on an earlier diagnosis of neurodegenerative disorders as evolving treatments are likely to be more effective before irreversible changes have occurred in the brain [1, 2]. The drive to seek early diagnostic clarification has led to an over 600% increase in referrals to secondary care memory clinics in the UK over the last ten years and generated considerable pressure on diagnostic pathways [3]. Although these dramatic changes have increased the number of patients in whom neurodegenerative disorders have been identified, a large proportion of the patients now referred to specialist memory clinics actually have functional (non-progressive) memory concerns without objective evidence of cognitive deficits. Therefore, improvements to stratification and screening procedures would be highly desirable and could enable better targeting of limited health care resources [4]. However, the early identification of patients with neurodegenerative disorders is a challenging task due to a lack of accurate predictive biomarkers suitable for routine screening or stratification. Biomarkers capable of identifying patients at high risk of developing the commonest cause of progressive cognitive decline, Alzheimer’s disease (AD), pre-symptomatically exist [5] but are either expensive and only available in very few centers (e.g. amyloid Positron Emission Tomography) or are invasive (e.g. amyloid and tau testing in the cerebrospinal fluid) and not suitable for for screening at the interface between primary and specialist care patients [6].

Tools for screening for AD exist, but do not work sufficiently well. For instance, the Dementia-Detection (DemTect) or the Montreal Cognitive Assessment (MoCA) are one-page screening tools that can be administered by a trained examiner in 10 minutes. However, both produce learning effects which limit the number of possible administrations. What is more, current cut-offs have poor specificity and have not been tested in people with FMD (who general practitioners may consider referring to specialist services because of their cognitive complaints). The ecological validity of these screening tests is also limited. Thus cheap, noninvasive and reliable stratification and screening tools which are fully automated, scalable, can be repeated and are remotely applicable are urgently required [4].

Recently, speech-based automatic detecting methods for mental illness have gained popularity. The speech analysis approach is a cheap and noninvasive and can be deployed remotely, making it it an attractive proposition for use in dementia assessment pathways as well. There has been a large increase in referrals from primary care of people with memory complaints to secondary care memory services, resulting in considerable pressure to diagnostic pathways [3]. Therefore improvements of stratification and screening procedures are crucial for health services to be able to cope efficiently with this trend [4]. When people visit a memory clinic, the assessment typically begins with a conversation with a specialist during which patients are asked a series of questions about their memory problems. This interaction provides important insights into the cognitive state of the patient. The clinician will note whether patient or accompanying others respond to questions, whether answers are quick and expansive or short and incomplete.

Several research studies have suggested different forms of speech analysis for the identification of dementia. Lopez-de-Ipena *et al*. [7, 8] examined a combination of linear and nonlinear acoustic features derived from spontaneous speech in a multi-lingual dataset of 70 participants. The features were used to build a machine learning model designed to capture the irregularities affecting speech caused by AD. König *et al*. [9] used recordings from three groups of subjects identified as AD (mean Mini-Mental State Examination (MMSE) score 19), healthy elderly controls (HC) (mean MMSE 29) and mild cognitive impairment (MCI, mean MMSE 26). The participants were instructed to carry out four short vocal cognitive tasks. For each task, a number of acoustic features were extracted and used to train a support vector machine (SVM) classifier. Three different identification scenarios were tested: HC vs MCI, HC vs AD and MCI vs AD with reported classification accuracies of 79% ±5%, 87% ± 3% and 80% ±5% respectively. Weiner *et al*. [10] built a Linear Discriminant Analysis (LDA) classifier to perform a multi-class experiment on longitudinally collected speech samples. The dataset recordings were a collection of biographic interviews and cognitive tests of 74 participants administered by psychiatrists during the course of three separate visits. Follow-up cognitive tests demonstrated that some participants changed from the healthy cognition group into the Aging-Associated Cognitive Decline (AACD) and AD groups. A total of 98 speech samples were analysed (HC n = 80, AACD n = 13, and AD n = 5). A set of acoustic features was extracted using manually transcribed files and voice activity detector software; these features included the mean of silent segments, speech and silence durations, silence to speech ratio, silence count ratio, word and phoneme rates and silence to word ratio. Using these variables, the LDA model achieved an classification accuracy of 85.7% between the three patient classes.

Jarrold *et al*. [11] distinguished between different types of dementia by combining lexical and acoustic feature profiles extracted from spontaneous speech. The features were collected from 9 controls and 39 patients who had been diagnosed with different dementia sub-types: fronto-temporal degeneration (mean MMSE 24), primary progressive non-fluent aphasia (mean MMSE 22), semantic dementia (mean MMSE 17) and AD (mean MMSE 18). The acoustic features included phoneme duration, speech rate, mean and standard deviation (STD) of the duration of consonants, vowels and pauses as well as the mean and STD for voice and voiceless duration segments. For the lexical features profile, they extracted the frequency occurrence of 14 different part of speech features (verbs, pronouns, nouns, function words, etc.). The feature-based profiles were extracted from structured interviews and used as input to a machine learning algorithm. A classification accuracy of 88% was achieved by using a multi-layer perceptron as a binary classifier to differentiate between the AD and HC groups exclusively, while the classification accuracy dropped to 61% when all participants (with different types and severity of ND) were included.

Toth *et al*. [12] proposed a speech-based method for the early diagnosis of AD. The approach was evaluated using German speech recordings from 84 participants performing three tasks: immediate and delayed recall, in addition to spontaneous speech. The mean MMSE scores for the MCI (n = 48) participants was 26.9 while the HC (n = 36) group had mean MMSE scores of 29.1. After watching two short films, the participants were instructed to talk about the contents of these movies, once immediately after the end of the first movie, and secondly, after a 1-minute delay or a distraction for the last one. The spontaneous speech task involved recordings of the participants describing their previous day. From participants’ speech prompted by these tasks, Toth et al. extracted a set of acoustic features measuring the number and average length of silent pauses and filled pauses, as well as rates for both speech and articulation. These features were used to build three classifiers to differentiate between MCI and HC. The best accuracy was achieved with a Random forest classifier with an F1 score of 78.8%.

Gosztolya *et al*. [13] examined the fusion of two SVM models to perform binary, and multi-class classification to classify between three subgroups of subjects HC(n = 25), MCI (n = 25) and AD(n = 25, mean MMSE scores 29, 27 and 23 respectively). The first model was built using a set of acoustic features which had been extracted using automatic speech recognition software (articulation rates, utterance length, silent and filled pauses, a ratio of pause and speech). The second model was built using linguistic features extracted from manually annotated transcripts and included the number and rate of adjectives, nouns, verbs, pronouns, conjunctions, uncertain words, content words and function words. These features were extracted from recordings of Hungarian spontaneous speech. The accuracy of the fused model varied between 80% for both HC vs. MCI and MCI vs. AD and 86% for HC vs. AD, while the accuracy for the multi-class task, i.e. HC vs. MCI vs. AD was 81%.

In our previous work [14], we demonstrated that acoustically-derived features could separate patients with AD (n = 167, mean MMSE 19) from HCs (n = 97, mean MMSE 29). Our previous analysis used a longitudinal dataset known as DementiaBank [15] which holds recordings of subjects carrying out the Cookie Theft Picture description task. The descriptions were recorded on a yearly basis, the first visit date varied between subjects from (1983-1988), the last 7^*th*^ visit was recorded by the participants still remaining in the study in 1996. A total of 263 features were extracted and reduced using a feature selection technique to a set of 20 best discriminating features. Four machine learning classification algorithms were built using the top 20 features, achieving a maximum classification accuracy of 94.7%. The acoustic features in [14], were further extended in our work in [16], for the same dataset, to develop a regression model capable of predicting the clinical Mini-Mental State Examination (MMSE) scores (0-30) measured longitudinally during three visits in the AD group. We achieved a mean absolute error of 2.6, 3.1 and 3.7 in the prediction of MMSE scores across the first, second and third visits respectively. A classification model was also proposed in [16] to distinguish between three groups of subjects (AD, MCI and HC) (with mean MMSE across the three visits of 21.1, 27.7 and 29.1 respectively), and we achieved accuracies ranging from 89.2% to 92.4% when doing pairwise classification between the AD, MCI and HC classes.

Recently, Mirheidari *et al*. [17–19] proposed an automatic method for the differentiation between patients with cognitive complaints due to ND (mean MMSE 18.8) or FMD (mean MMSE 28.9) inspired by diagnostic features initially described using the qualitative methodology of Conversation Analysis [20, 21]. In their work, a set of linguistic, acoustic and visual-conceptual features were extracted and used to train a number of classifiers. The highest classification accuracy of 97% was achieved using a linear support vector model. In the current work, we propose an automatic method to discriminate ND from FMD based solely on acoustic analysis of the same conversations used in [17].

The work presented here differs from the approach pursued in the previous studies by Mirheidari et al. both in terms of complexity and in terms of the acoustic characteristics used. Here, we explore an acoustics-only approach based on data directly extracted from patients’ speech signal. The prior study relied on more complex features (including features based on the contributions of clinicians and carers to the interaction) and required automatic speech recognition, natural language processing and natural language understanding.

## Materials and methods

### Participants

The dataset used in this experiment was recorded as part of a study conducted in the neurology-led memory clinic at the Royal Hallamshire Hospital in Sheffield, United Kingdom. Participants were recruited between October 2012 and October 2014 and the initial consultations between the neurologists and patients in the memory clinic were video and audio recorded. The study was approved by the National Research Ethics Service (NRES) Committee Yorkshire & The Humber—South Yorkshire. All participants were sent an information sheet prior to taking part in the study and given an opportunity to ask questions. They all gave written informed consent to participate and were informed that they could withdraw from the study at any time. Patients consented to their data being used for additional analyses by the research team but not to recordings of their interactions being made publicly available. All patients were referred because of memory complaints by General practitioner or other hospital consultant. Participants were encouraged to bring a companion such as carer or family member (if available) along to their appointment. Further details about the participant selection procedure have been provided previously [17]. At the clinic, patients underwent a clinical assessment by a neurologist specialising in the diagnosis and treatment of memory disorders. In addition, all underwent the Addenbrooke’s Cognitive Examination-Revised (ACE-R) cognitive assessment [22]. Neurologists also screened patients for clinical evidence of depression. Patients thought to be depressed clinically or patients with PHQ-9 [23] scores indicating a high risk of clinical depression were excluded from this study [17]. All participants also completed the Generalised Anxiety Disorder (GAD7) questionnaire [24] although high levels of anxiety were not considered an exclusion criterion. The final diagnoses of ND or FMD were formulated by Consultant Neurologists specialised in the treatment of cognitive disorders and also took account of brain Magnetic Resonance Imaging (MRI) findings and the result of a detailed separate neuropsychological assessment including the MMSE [25]; tests of abstract reasoning [26]; tests of attention and executive function [27]; category and letter fluency; naming by confrontation and language comprehension [28]; and tests of short and long-term memory (verbal and non-verbal) [29]. Table 1 gives an overview of particpants’ details and test scores. The ND group consisted of 10 cases of AD, 2 amnestic MCI, 2 BvFTD and one vascular dementia.

**Table 1:** Participants’ details and test scores.

	FMD(n = 15)	ND(n = 15)	Cut off	Max score	P-value
**Age**	57.8± 2.0	63.7 ± 2.3	N/A	N/A	p = 0.06
**Female**	60%	53%			ns*
**ACE-R**	93.0 ± 1.4	58 ± 5.21	88	100	p < 0.0001
**MMSE**	28.9 ± 0.2	18.8 ± 2.0	26.3	30	p < 0.0001
**PHQ9**	5.6 ± 1.0	5.3 ± 2.0	5	27	ns
**GAD7**	4.7 ± 1.2	4.8 ± 1.5	5	21	ns
**History taking part in minutes**	range (10.1-32.3)	range(7.3-29.0)			

ACE-R: Addenbrooke’s Cognitive Examination-Revised; MMSE: Mini-Mental State Examination; PHQ9: Patient Health Questionnaire-9; GAD-7: Generalised Anxiety Assessment 7. Unpaired T-test was used. ns* = not significant

### Diagnosis process

Diagnoses of FMD were based on the criteria formulated by Schmidtke *et al*. [30] (although the proposed maximum age cut-off proposed by Schmidtke *et al*. was not applied). The participants in the ND group received the following neurological diagnoses: amnestic MCI as described by Petersen *et al*. [31], behavioral variant frontotemporal dementia as defined by Rascovsky *et al*. [32], and Alzheimer’s disease diagnosis according to the NINCDS-ADRDA criteria [33].

### Memory clinic instructions

The neurologists, whose conversational activities elicited the dataset used in this study, followed a communication guide (developed on the basis of previously observed routine practice). Doctor-patient encounters began with a history-taking phase, which was followed by a brief cognitive examination (e.g. ACE-R), Table 2 provides timing details for the clinical sessions, history taking and percentage of the patients’ contribution. Neurologists were encouraged to ask open questions to prompt conversation from the person with memory complaints. Examples of these questions includes the following: “When did your memory last let you down?”, “Who is the most concerned about your memory—you or somebody else?”, “Tell me a bit about yourself, where did you go to school?”, “What did you do after you left school?”, “Who looks after your finances?”, “Do you smoke, have you ever smoked in the past?”, “Why have you come to clinic today and what are your expectations?”.

**Table 2:** Details of the clinical session times expressed in minutes.

		Clinical session (Conversation +verbal fluency test)	Conversation part only	Patient contribution to the conversation
**Mean time ± STD**	FMD	34.3 ± 9.9	17.9± 8.5	11.5± 6.3
	ND	39.2 ± 8.0	19.4± 7.0	6.2± 4.5
**Range time**	FMD	(22.3–52.4)	(10.1–32.3)	(5.3–26.5)
	ND	(24.7–57.0)	(7.3–29.0)	(1.1–15.5)
**Percentage**	FMD	Not applicable	50.9 %	63.0 %
	ND	Not applicable	49.79 %	32.4 %

STD: Standard deviation

## Description of acoustic-only dementia detection system

**Fig 1:**
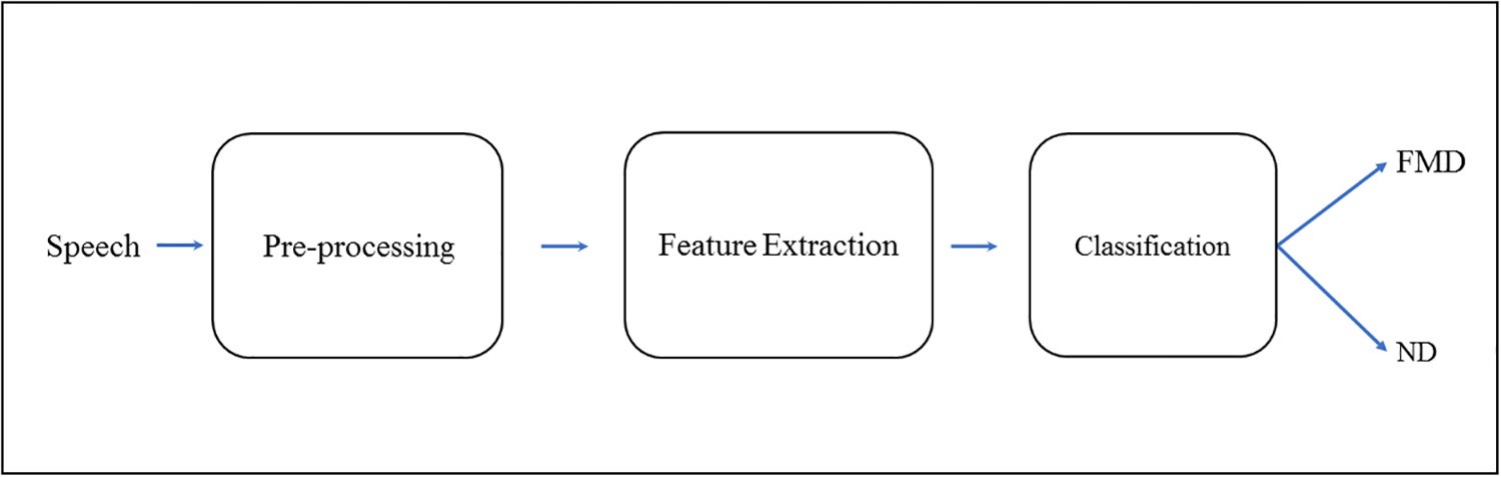
The acoustic-only system for the identification of patients with neurodegenerative cognitive complaints.

The system is intended as an early stratification tool for patients presenting with progressive ND-related cognitive problems based solely on diagnostic acoustic features in patients’ speech. As illustrated in Fig 1, it consists of three main stages: pre-processing, feature extraction and machine learning based classification.

### Pre-processing

The clinical sessions were recorded using a “ZOOM H2N” portable digital recorder. The device was placed on the table between patient and doctor (within 1 m of neurologist, patient and accompanying person). The device produced audio files in “MP3” format with a sampling frequency of 44.1 kHz. The speech recordings were first converted to “wav” format and down sampled to 16kHz, and then inspected for background noise which may affect the quality of the extracted features. We used the Audacity(R) software [34] for both the audio conversion and denoising process in which we applied the spectral noise gating method. Before extracting any features from the recordings, we identified and isolated the segments of the conversations containing the patient’s utterances (i.e. we excluded those by the neurologist and any companions). For this study, we used the manually extracted turns as marked in the transcribed text files associated with the audio recordings.

### Feature extraction

The aim of this work was to explore the potential of using only acoustic features to differentiate between FMD and ND. The authors of [17] applied a limited set of acoustic features inspired by the previous qualitative Conversation Analysis (mainly statistics of speech and silence). In the present study, we used a larger set of acoustic features, which we used previously to differentiate between AD, MCI and healthy elderly subjects on the DementiaBank data set [16]. These features can be grouped into Speech and silent features, phonation and voice quality features, and spectral features. Below, each main acoustic feature type is described in detail (see Table 3 for a summary of all features).

**Table 3:** Acoustic features.

Features	Type	Number of features
Speech and silent statistics	Speech and silent features	15
Fundamental frequency (F0)	Phonation and voice quality	3
Harmonic-to-noise ratio (HNR)	Phonation and voice quality	3
Noise-to-harmonic ratio (NHR)	Phonation and voice quality	3
Shimmer scales	Phonation and voice quality	3
Jitter scales	Phonation and voice quality	3
Number of voice breaks	Phonation and voice quality	3
Degree of voice breaks	Phonation and voice quality	3
Mel frequency cepstral coefficients (MFCC)	Spectral features	5
Filter bank energy coefficient (Fbank)	Spectral features	5
Spectral Subband Centroid (SSC)	Spectral features	5
Total		51

#### Speech and silent features

The frequency and duration of pauses has previously been reported to be of great value in the detection dementia by means of speech analysis. In particular, it has been found that the speech of people with dementia is disrupted by more pauses compared to that of healthy people [9, 35–38]. Based on this, we expected that ND patients would produce more and longer pauses, with shorter and fewer utterance periods compared to those with FMD. We considered a silent segment of ≥ 0.25 seconds as a pause and a minimum voice segment of 0.5 seconds as a speech segment. We used the Praat [39] software to identify pauses and speech segments in the recordings. A total of 15 features were used in the study including the average response time, the max, mean and STD of the pauses and speech segments, the ratios of both max pause and speech segments and total time, the ratio of max speech and max pause segment. The last three features were the mean, STD, and variance of speech segments ≥ 0.8 seconds (i.e., excluding the filler words).

#### Phonation and voice quality features

Phonation and voice quality features were included because previous studies found them to be predictive of ND diagnoses. Meilán *et al*. [40] and Lopez-de-Ipena *et al*. [41] achieved accuracies of 84.8% and 96.9% respectively when classifing between AD and HC subjects using these acoustic characteristics. This group of features includes the fundamental frequency (F0) and its related variances (shimmer and jitter). The F0 is a measurement of vocal fold oscillations [42] that are known to be nearly periodic in healthy voices, but less so in voice pathologies [43, 44]. Jitter_(*local*)_ describes the frequency alteration from cycle to cycle, while the shimmer_(*local*)_ measures the amplitude fluctuations of the consecutive cycles. [39] provides further details of these parameters.

The voice quality parameters included; the harmonic-to-noise ratio (HNR) which measures how much energy there is in the periodic part of the signal compared to its non-periodic part; and noise-to-harmonic ratio (NHR) which measures the amplitude of the noise generated due to incomplete closure of the vocal folds during the production of the speech relative to tonal components [45]. Furthermore, we included the; number of voice breaks (distances between pulses greater than 16 milliseconds); degree of voice breaks (ratio of the breaks’ total duration to the total duration of the analysed signal) [39]. The feature vector for this group contains 21 features, generated by estimating the mean, STD and variance of the previously mentioned features.

#### Spectral features

Speech production involves the movement of articulators including the tongue, jaws, lips, and other speech organs. The position of the tongue plays a key role in creating resonances in the mouth. Although speech articulation is relatively preserved in the commonest types of ND, it is conceivable that ND could have measurable effects on the coordinated activity of speech articulators and thereby spectral features. Mel frequency cepstral coefficients (MFCCs) [46] were extracted to capture the spectral content of the speech signal. We hypothesised that patients in the ND group might be characterised by lower spectral coefficients valued than those in the FMD group. The MFCCs aims to compute the energy variations between frequency bands of a speech signal. MFCCs have become widely used in speaker verification, speech recognition and for the extraction of paralinguistic information since they were proposed by Davis and Mermelstein back in 1980. We follow the standard method for extracting the MFCCs, [47], in which, each 25ms frame is converted into the frequency domain using the fast Fourier transform (FFT) before the power spectrum is estimated by taking the absolute value of the complex FFT and squaring the result. Next, the Mel triangular filter-banks are applied and calculated by converting the frequencies into the Mel scale and summing the energy for each filter. By taking the logarithm of all filter-bank energies we obtain our second set of features, namely the logarithmic energy of the Mel filters (Fbanks; 26 features).

The last set of SF features were the spectral sub-band centroids (SSCs). SSCs aims to locate the spectrum centre of mass and found to be valuable in measuring the cognitive load le *et al*. [48]. We therefore hypothesised that SSCs could be useful in this study, and a total of the first 13 coefficients were included, SSCs are extracted by dividing the energy in each filter-bank (i.e., 26 filter-banks as estimated previously) by the total energies of all filter-banks [49, 50].

#### Statistical descriptive features

The dataset used in this study comprised of the part of routine outpatient encounters in which the doctor took the patient’s history, the interactions lasted 18 minutes on average (see Table 1). The duration of the recordings allowed for the use of long-term features based on statistics calculated for this part of the conversation. Since the spectral coefficients are generated for each frame of all utterances, we first estimated their mean per utterance, and then we weighted them by dividing the averaged coefficients by the utterance time, and that produced the averaged weighted spectral coefficients (AWSC). The motivation behind that is the ND subjects are likely to provide fewer responses compared to FMD, so we believe that incorporating time factor will improve the predictive ability of the spectral features and hence increases the system accuracy. The statistical descriptive features are the mean, STD, min, max and the variance applied to each subgroup of the AWSC (i.e., MFFCs, Fbanks, and SSCs) resulting in adding an extra 15 features which makes the total number of features used in this experiment 51.

### Feature selection

Feature selection (FS) is the process of selecting a subset of original features in order to optimally reduce the feature space according to a certain evaluation criterion [51, 52]. This process is considered an effective way to remove those features which have a negative impact on the learning process thereby leading to faster learning time and increased model accuracy while reducing complexity, as the model is trained using a smaller set of features [53].

In general, there are three feature selection techniques namely the filter, wrapper and embedded [51, 52, 54]. The filter approach uses a specific ranking criterium (for example the Pearson correlation coefficient) to generate scores for each feature. The main advantages of the filter method are the low computational cost and speed compared to the wrapper approach; however, the feature ranking is done independently of the model’s predictive ability, and that often leads to a loss of performance. The wrapper technique is computationally more expensive and slower compared to the filter approach. However, the wrapper usually results in improved performance because it utilizes the model’s ability to rank and select the best subset of features. Further, the wrapper method applies a learning algorithm known as the evaluator, and a search technique to find the combination achieving maximum model performance. The embedded method, on the other hand, executes the feature selection as part of the learning procedure, for instance, tree classifiers have a built-in feature importance identification capability and can therefore select the best subset of features.

We used a wrapper method based on an SVM evaluator known as the recursive feature elimination (RFE) technique [55], in which, the features are eliminated sequentially and the model performance estimated each time until all features have been excluded. The feature that has the maximum negative impact on the result is considered to be the most important one. Likewise, the rest of the features are then ranked. When using tree classifiers (random forest and Adaboost) we utilised the built-in feature selection.

Another way of selecting features is by performing a statistical analysis, which measures the significance level of the mean difference between the FMD/ND classes. The null hypothesis assumes that there is no significant difference between the means for a particular feature and hence, that feature should be ignored. On the other hand, features that reject the null hypothesis are selected. For this task, we used the SPSS software [56] to perform Mann-Whitney u-tests appropriate for non-parametric data. Table 4 shows the best subset of features selected using both RFE and the embedded approaches as well as the corresponding statistical U-test and p-values (below the 0.05 significance level) at a 95% confidence interval.

**Table 4:** Top (22) selected features using the wrapper, embedded and their statistical U-test.

Rank	Features	U	P	Rank	Features	U	P
1	Mean time of all speech segments excluding filler words	17.0	0.00007	12	Mean response time	42.0	0.004
2	Ratio of max speech segment to the max pause time	19.0	0.0001	13	VAR degree of voice breaks	44.0	0.004
3	STD of total speech segments time excluding filler words	19.0	0.0001	14	STD of number of voice breaks	44.5	0.004
4	STD of the speech segments time	20.0	0.0001	15	Mean degree of voice breaks	45.5	0.005
5	VAR of total speech segments time excluding filler words	20.0	0.0001	16	Mean of Fbank coefficients	49.0	0.008
6	Ratio of max pause time to the total turn time	24.0	0.0002	17	Min of Fbank coefficients	49.5	0.009
7	Ratio of total pauses time to the total turn time	24.5	0.0002	18	VAR of SSC coefficients	52.5	0.01
8	Ratio of total speech segments time to the total turn time	26.0	0.0003	19	STD of SSC coefficients	59.5	0.02
9	VAR of number of voice breaks	26.0	0.0003	20	STD of MFCC coefficients	60.0	0.03
10	Ratio of total No. of pauses to the total turn time	27.0	0.0003	21	VAR of Fbank coefficients	60.5	0.03
11	Mean number of voice breaks	30.0	0.0006	22	Mean of MFCC coefficients	63.0	0.04

U: Mann-Whitney u-tests.

Sample size *n*_1_ = *n*_2_ = 15.

## Validation scheme

**Fig 2:**
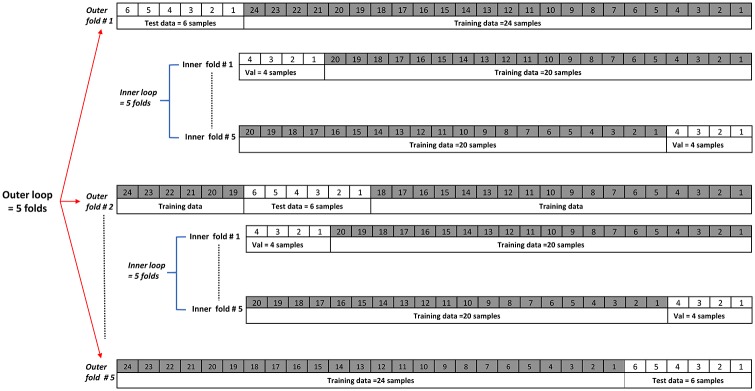
Nested k-fold cross validation with K = 5.

In the machine learning community, cross-validation is widely used to ensure an effective method of model selection to achieve, a robust performance evaluation and prevent over-fitting [57]. We used K-fold cross-validation with k = 5, to partition the data into five equal parts called “folds”. The model was trained using four out of five folds and tested with the remaining 5^*th*^ fold. This step was repeated k = 5 times until all folds had been used in the training and testing process. This however, did not generate the validation set directly. Instead, we used what is known as the nested k-fold cross-validation method, which uses two k-fold loops namely the outer and the inner loops. The outer loop generates the testing (1/5 data) and the training (4/5 data) folds, while the inner loop takes all the training folds combined (coming from the outer loop) and generates the validation and training folds. Fig 2 shows the design of the nested 5-fold cross-validation. Feature selection and the model’s hyper-parameter tuning were explored and the model with the best features and best parameters was tested using the test folds. This process runs through all the loops and the final model result is reported as the average of the best model’s scores across the outer test folds. Importantly, each fold generated had to contain a balanced number of samples between the two classes for the nested k-folds cross-validation in order not to skew the output towards one class. We used Scikit learn libraries to perform this task [55].

We also explored another scenario, in which we increased the sample size from 30 to 230 samples by partitioning each recording into 1-minute segment lengths, and extracted all features as mentioned in the previous sections. In this way the results were evaluated using a larger dataset, however, the balance between the two classes was lost due to the fact that the talk captured from conversations in the FMD group was longer compared to that from the ND group (i.e. 60% of the 230 samples were from the FMD group). We used the same principle of nested cross-validation as explained before in Fig 2, but changed from the k-fold method to a leave one group out cross validation (LOGOUT). The LOGOUT method guarantees that the group of samples that belong to one patient will always be in the same fold, for example, all segments from recording number 1 will be in the training data. LOGOUT also differed from the k-fold in generating the partitions. In LOGOUT the training data will be (G-1), and the test data will be the last remaining (G), (G) is the number of groups (30 in our case), this will loop again but now 30 times instead of five as in k-fold. The feature selection, model hyper-parameter tuning, the best model selection procedure, and the reported results remained the same as those calculated by the procedure outlined earlier. One important step we included before training any models is the feature normalization. This preprocessing step was executed at the training phase (training data) and excluded from the validation and test phases. Different methods can be used for feature normalization. We used equation Eq (1) which is known as the standard scalar score for the training sample x given by:

$$\begin{array}{c} Stand\left(X\right)=\frac{x-\mu }{\sigma } \end{array}$$ (1)

Where *μ* and *σ* is the mean and standard deviation of the training samples respectively.

## Results

The results of the study suggest that machine learning models based on the analyses of the acoustic data from patients with cognitive complaints are capable of detecting differences between the two classes, ND and FMD, in keeping with prior research [7–9, 14, 16]. We explored the discriminating potential of acoustic features using five different classification algorithms (SVM, random forest, Adaboost, multi-layer perceptron, and SGD) and tested our findings using the validation procedure described above. The best models’ results are listed in Tables 5 and 6. These were obtained using both scenarios: the original dataset with 30 samples and the augmented dataset with 230 samples. The average results for all models improved regardless of feature selection method and for both Tables 5 and 6. All models scored 97% accuracy except Adaboost which reached a maximum at 93% when the original dataset of 30 recording samples was used. The number of features used for each model is smaller when the wrapper and embedded approaches are used compared to the statistical ranking approach, for example the SVM wrapper model needed only 9 out of 22 ranked features from Table 4 compared to 11 features when the ranking is performed based on statistical significance. The differences between the two feature selection approaches were expected because both methods utilised the classifier scores to identify the best set of features maximizing the performance while the analytical approach, on the other hand, included an un-optimized set of features for the models to reach their maximum accuracies.

**Table 5:** First scenario classification accuracies under different feature subsets and classifiers.

Classifier	Accuracy using All features (51)	Accuracy with feature selection	No. of selected features	Accuracy using features with significance statistical P-value	No. of selected features
**Linear SVM**	**93.0** ± 0.16 %	**97.0** ± 0.13 %	9	**97.0** ± 0.13 %	11
**Random forest**	90.0 ± 0.27 %	**97.0** ± 0.13 %	11	**97.0** ± 0.13 %	15
**Adaboost**	85.0 ± 0.40 %	93.0 ± 0.16 %	11	90.0 ± 0.24 %	21
**MLP**	**93.0** ± 0.16 %	**97.0** ± 0.13 %	20	**97.0** ± 0.13 %	22
**Linear via SGD**	90.0 ± 0.16 %	**97.0** ± 0.13 %	14	**97.0** ± 0.13 %	21
**Mean**	**90.2 %**	**96.2 %**	13	**95.6 %**	18

**Table 6:** Classification accuracies for the second scenario (augmented dataset).

Classifier	Accuracy using All features(51) ± STD	Accuracy with feature selection ± STD
**Linear SVM**	**87.0** ± 0.12 %	**92.0** ± 0.18 %
**Random forest**	84.0 ± 0.23 %	88.0 ± 0.28 %
**Adaboost**	81.0 ± 0.26 %	87.0 ± 0.29 %
**MLP**	**86.0** ± 0.26 %	91.0 ± 0.14 %
**Linear via SGD**	**87.0** ± 0.11 %	**92.0** ± 0.16 %
**Mean**	**85.0 %**	**90.0 %**

Table 6 shows the models’ results when evaluated using the second scenario dataset. Both the SVM and SGD models score 92.0%, on average the five models achieved 90% which is less compared to 96.2% average results for the first scenario. The difference between the results was expected because the five models of the second scenario were evaluated on much more data samples, so the models’ error percentage is likely to increase, however, this result may be considered more realistic and generalized thus perform better in future deployment.

## Discussion

This study has shown that automatic speech analysis technology focusing and acoustic features in their speech could be a valuable complementary method in the diagnostic pathway of patients presenting with cognitive concerns. We aimed to build a machine learning model that learns from our data and is able to predict the cognitive status of patients referred to a specialist memory clinic. In this study we used a binary classification; FMD or ND. The highest classification accuracy reached 97% which was achieved by four machine learning diagnostic models: SVM, random forest, multi-layer perceptron, and SGD. The most discriminant features utilized by the models include ratios and statistics of pauses and utterances, which aligns with the literature. ND patients’ speech has previously been found to be characterized by an increased number and duration of pauses as well as a reduction in the number of utterances which may be caused by difficulty in word finding (lexical retrieval) [36]. Similarly Singh *et al*. [58] and Roark *et al*. [35] reported that the mean time of both pauses and speech are useful in discriminating healthy subjects from MCI and AD patients. Other features of discriminating value in our study included the number and degree of voice breaks, which aligns with findings also previoulsy reported by Meilán *et al*. [40].

Further, the importance of the spectral features including the average of Fbank and MFCC coefficients and the standard deviation of MFCC and SSC coefficients. These features measure the energy variations between frequency bands of a speech signal. As such they are harder to interpret in the context of detecting neurodegenerative cognitive decline. However, these features also capture some articulatory information expressed by lower resonance in the vocal tract, a prominent finding in ND patients (Fraser *et al*. [59] and in our previous works in [14, 16]). Some of the acoustic features we examined in this study were excluded from the discriminatory models finally created because they did not differe significantly between the two groups. These features include the fundamental frequency, shimmer, jitter and harmonic to noise ratio which appears to be in contrast to what have been stated in [40]. This may be because these features examine characteristics of the speech not strongly affected by FMD and ND, unlike in Parkinson’s disease, where dysphonia is a common and key clinical feature [45, 60].

Comparing our approach to that used in the previous study using the same dataset [17], the currently proposed method, based exclusively on acoustic findings, is computationally much less demanding than an analysis based on a combination of acoustic, lexical, semantic and visual-conceptual features. Furthermore, the previous classification approach included input features from the neurologist, and from accompanying persons whereas the present study only uses utterances from patients themselves. Although only patients’ contributions and the analysis of acoustic signals were used in the present study, the overall classification accuracy improved to that achieved using the more complex approach (proposed 96.2% vs 95.0% [17]).

The sensitivity and specificity of the proposed system were 93.75% and 100% respectively. This compares well with other dementia screening modalities, for example, electroencephalography (EEG) tests which may be relatively cheap, noninvasive and widely available, but are still rather cumbersome, and, more importantly, only have a sensitivity of 70% for the detection of early Alzheimer’s disease [61]. Positron Emission Tomography (PET), although associated with much higher sensitivity and specificity (both at 86.0%) is invasive, requires the injection of a radioactive tracer via a peripheral cannula, means that patients have to be fasting for four hours before the test and is very costly [62]. Single photon Emission Computed Tomography (SPECT) is another diagnostic tool capable of demonstrating changes early in the course of neurodegenerative disorders with high sensitivity (86.0%) and specificity (96.0%), but is as cumbersome and costly as PET and also exposes patients to a high dose of radiation [63].

How does the proposed method compare to currently available approaches for screening at the interface between primary and specialist care? The test most commonly used world wide is the Mini Mental State Examination (MMSE). It takes and average of 10 minutes to administer. Although the MMSE has high sensitivity (87.3%) and specificity (89.2%) scores it is not sufficiently sensitive in the early stages of dementia. What is more, it is influenced by patients’ level of education [64]. The ACE-R requires 12-20 minutes to administer and performs at a similar level (sensitivity 94.0% and specificity 89.0%), however, it does not provide feedback on why a particular diagnosis may have been made [65]. The clock drawing test (CDT), despite taking only 2-3minutes to complete and having impressive screening performance (sensitivity 92.8% and specificity 93.5%), is not frequently used as it does not test memory as such, which especially limits its usefulness in AD. What is more, the scoring may be tricky due to having 8 different assessment settings. The test is also not suitable for people with illiterate patients who can’t perform paper and pencil tests [66].

Importantly, all tools described above are the state of the art in dementia screening and currently utilized worldwide. In contrast, our approach has, so far, only been tested on a small dataset, so its performance should not be generalized unless been validated with very large dataset. Having said that, the study highlight the significance of an acoustic-only based approach as a promising low cost diagnostic aid in assessment pathways of patients presenting with cognitive problems. In view of the relatively low hardware (microphone) and computational complexity this system could easily be deployed in settings other than memory clinics. This may be desirable from a patients’ perspective and more cost-effective from the perspective of health care providers. Moreover, whereas the application of a system based on semantic understanding is limited to the language(s) it was trained to interpret, the present system, using acoustic features only, is much less dependent on the particular language used by a patient and should therefore be more widely generalisable.

There are several limitations to this study. Firstly the data set is relatively small with only 15 cases in each group. However, we did model a larger dataset, and the resulting difference was only a 5% decrease in performance. However, the size difference between the two scenarios was more > 750% (30 samples compared to 230) i.e. for each 100% increment in size the performance decreased by 0.67% which shows that the proposed approach did not deviate badly. Also the high accuracy of this model reflects the difference between the two groups; notably the ND group were mostly in the moderate stages of ND, with a mean MMSE of 18.8(+/−2.0). Furthermore, this approach was based on manually annotated files that identified the patients’ turns in standardized conversations, however, this limitation can be overcome by using an automatic speaker diarization software which is capable of providing information about who is speaking and when. Further work is required including evaluating our model’s performance with spontaneous conversations and with the use of speaker diarization software to fully automate the proposed system.

Future directions should also investigate larger numbers of participants with MCI who are in the earlier stages of AD [67] and aim to correlate noninvasively collected disease markers into an established tool for patients with cognitive concerns [5]. Additionally we will examine the performance of the proposed system in the prediction of clinical memory test scores such as MMSE and ACE-R. Future work will also focus on the wider applicability of the proposed feature set, both when dealing with more diagnostic categories (including patients with depression-associated memory problems) and when evaluating larger collections of recordings of patients with different forms of ND and longitudinal data collection to monitor patients for instance as an objective marker of treatment effects or the purpose of ensuring a patient’s continuing safety.

## Conclusions

The results of this study lead to several conclusions. First, a relatively small number of extracted acoustical features are shown to be of great importance in the differentiation between ND and FMD. These features are likely related to changes in the neurobiology associated with a given neurodegenerative cognitive disorder, reflected in the acoustic output. Secondly, the proposed approach can be easily deployed at clinics and during standard clinical encounters. This will require only minimal effort on the part of the examiner and mean a much quicker diagnosis for the examinee. Finally, despite the limitations of this study, our findings show that acoustic-only features offer a potential low-cost, simple and alternative to more complex features requiring automatic speech recognition, part-of-speech parsing, and understanding of speech in the automated screening or stratification of patients with cognitive complaints. Hence it has great potential for use early in pathways that assess people with cognitive complaints [4, 68].

## Supporting information

S1 File Dataset point of contact. (PDF)

S2 File Patient consent form. (PDF)

## References

[1] Blackburn DJ, Wakefield S, Shanks MF, Harkness K, Reuber M, Venneri A. Memory difficulties are not always a sign of incipient dementia: a review of the possible causes of loss of memory efficiency. British Medical Bulletin. 2014;112(1):71–81. 10.1093/bmb/ldu029 25274571

[2] Commissaris C, Ponds R, Jolles J. Subjective forgetfulness in a normal Dutch population: possibilities for health education and other interventions. Patient Education and Counseling. 1998;34(1):25–32. 10.1016/S0738-3991(98)00040-8 9697554

[3] Hodge S, Hailey E. English national memory clinics audit report. London: Royal College of Psychiatrists. 2013.

[4] Laske C, Sohrabi HR, Frost SM, López-de Ipiña K, Garrard P, Buscema M, et al Innovative diagnostic tools for early detection of Alzheimer’s disease. Alzheimer’s & Dementia: the Journal of the Alzheimer’s Association. 2015;11(5):561–578. 10.1016/j.jalz.2014.06.004 25443858

[5] Dubois B, Feldman HH, Jacova C, Hampel H, Molinuevo JL, Blennow K, et al Advancing research diagnostic criteria for Alzheimer’s disease: the IWG-2 criteria. The Lancet Neurology. 2014;13(6):614–629. 10.1016/S1474-4422(14)70090-0 24849862

[6] Metternich B, Schmidtke K, Hüll M. How are memory complaints in functional memory disorder related to measures of affect, metamemory and cognition? Journal of Psychosomatic Research. 2009;66(5):435–444. 10.1016/j.jpsychores.2008.07.005 19379960

[7] López-de Ipiña K, Alonso JB, Travieso CM, Solé-Casals J, Egiraun H, Faundez-Zanuy M, et al On the selection of non-invasive methods based on speech analysis oriented to automatic Alzheimer disease diagnosis. Sensors. 2013;13(5):6730–6745. 10.3390/s130506730 23698268 PMC3690078

[8] López-de Ipina K, Solé-Casals J, Eguiraun H, Alonso JB, Travieso CM, Ezeiza A, et al Feature selection for spontaneous speech analysis to aid in Alzheimer’s disease diagnosis: A fractal dimension approach. Computer Speech & Language. 2015;30(1):43–60. 10.1016/j.csl.2014.08.002

[9] König A, Satt A, Sorin A, Hoory R, Toledo-Ronen O, Derreumaux A, et al Automatic speech analysis for the assessment of patients with predementia and Alzheimer’s disease. Alzheimer’s & Dementia: Diagnosis, Assessment & Disease Monitoring. 2015;1(1):112–124. 10.1016/j.dadm.2014.11.012 PMC4876915 27239498

[10] Weiner J, Herff C, Schultz T. Speech-Based Detection of Alzheimer’s Disease in Conversational German. In: INTERSPEECH; 2016. p. 1938–1942.

[11] Jarrold W, Peintner B, Wilkins D, Vergryi D, Richey C, Gorno-Tempini ML, et al. Aided diagnosis of dementia type through computer-based analysis of spontaneous speech. In: Proceedings of the Workshop on Computational Linguistics and Clinical Psychology: From Linguistic Signal to Clinical Reality; 2014. p. 27–37.

[12] Tóth L, Hoffmann I, Gosztolya G, Vincze V, Szatlóczki G, Bánréti Z, et al A speech recognition-based solution for the automatic detection of mild cognitive impairment from spontaneous speech. Current Alzheimer Research. 2018;15(2):130–138. 10.2174/1567205014666171121114930 29165085 PMC5815089

[13] Gosztolya G, Vincze V, Tóth L, Pákáski M, Kálmán J, Hoffmann I. Identifying Mild Cognitive Impairment and mild Alzheimer’s disease based on spontaneous speech using ASR and linguistic features. Computer Speech & Language. 2019;53:181–197. 10.1016/j.csl.2018.07.007

[14] Al-Hameed S, Benaissa M, Christensen H. Simple and robust audio-based detection of biomarkers for Alzheimer’s disease. In: 7th Workshop on Speech and Language Processing for Assistive Technologies (SLPAT); 2016. p. 32–36.

[15] MacWhinney B. The talkbank project In: Creating and digitizing language corpora. Springer; 2007 p. 163–180.

[16] Al-Hameed S, Benaissa M, Christensen H. Detecting and predicting alzheimer’s disease severity in longitudinal acoustic data. In: Proceedings of the International Conference on Bioinformatics Research and Applications 2017. ACM; 2017. p. 57–61.

[17] Mirheidari B, Blackburn D, Harkness K, Walker T, Venneri A, Reuber M, et al Toward the automation of diagnostic conversation analysis in patients with memory complaints. Journal of Alzheimer’s Disease. 2017;58(2):373–387. 10.3233/JAD-160507 28436388

[18] Mirheidari B, Blackburn D, Reuber M, Walker T, Christensen H. Diagnosing people with dementia using automatic conversation analysis. In: In Proceedings of Interspeech. ISCA; 2016. p. 1220–1224.

[19] Mirheidari B, Blackburn D, Harkness K, Walker T, Venneri A, Reuber M, et al. An avatar-based system for identifying individuals likely to develop dementia. In Proceedings of Interspeech. 2017; p. 3147–3151.

[20] Jones D, Drew P, Elsey C, Blackburn D, Wakefield S, Harkness K, et al Conversational assessment in memory clinic encounters: interactional profiling for differentiating dementia from functional memory disorders. Aging & Mental Health. 2016;20(5):500–509. 10.1080/13607863.2015.1021753 25803169

[21] Elsey C, Drew P, Jones D, Blackburn D, Wakefield S, Harkness K, et al Towards diagnostic conversational profiles of patients presenting with dementia or functional memory disorders to memory clinics. Patient Education and Counseling. 2015;98(9):1071–1077. 10.1016/j.pec.2015.05.021 26116418

[22] Larner A. Addenbrooke’s Cognitive Examination-Revised (ACE-R) in day-to-day clinical practice. Age and Ageing. 2007;36(6):685–686. 10.1093/ageing/afm112 17881421

[23] Kroenke K, Spitzer RL, Williams JB. The phq-9. Journal of General Internal Medicine. 2001;16(9):606–613. 10.1046/j.1525-1497.2001.016009606.x 11556941 PMC1495268

[24] Spitzer RL, Kroenke K, Williams JB, Löwe B. A brief measure for assessing generalized anxiety disorder: the GAD-7. Archives of Internal Medicine. 2006;166(10):1092–1097. 10.1001/archinte.166.10.1092 16717171

[25] Folstein MF, Folstein SE, McHugh PR. “Mini-mental state”: a practical method for grading the cognitive state of patients for the clinician. Journal of Psychiatric Research. 1975;12(3):189–198. 10.1016/0022-3956(75)90026-6 1202204

[26] Raven JC. Guide to using the coloured progressive matrices. HK Lewis & Co; 1958.

[27] Stroop JR. Studies of interference in serial verbal reactions. Journal of Experimental Psychology. 1935;18(6):643 10.1037/h0054651

[28] De Renzi E, Faglioni P. Normative data and screening power of a shortened version of the Token Test. Cortex. 1978;14(1):41–49. 10.1016/S0010-9452(78)80006-9 16295108

[29] Wechsler D. Wechsler adult intelligence scale–Fourth Edition (WAIS–IV). San Antonio, Texas: Psychological Corporation 2014.

[30] Schmidtke K, Pohlmann S, Metternich B. The syndrome of functional memory disorder: definition, etiology, and natural course. The American Journal of Geriatric Psychiatry. 2008;16(12):981–988. 10.1097/JGP.0b013e318187ddf9 19038897

[31] Petersen RC, Caracciolo B, Brayne C, Gauthier S, Jelic V, Fratiglioni L. Mild cognitive impairment: a concept in evolution. Journal of Internal Medicine. 2014;275(3):214–228. 10.1111/joim.12190 24605806 PMC3967548

[32] Rascovsky K, Hodges JR, Knopman D, Mendez MF, Kramer JH, Neuhaus J, et al Sensitivity of revised diagnostic criteria for the behavioural variant of frontotemporal dementia. Brain. 2011;134(9):2456–2477. 10.1093/brain/awr179 21810890 PMC3170532

[33] Albert MS, DeKosky ST, Dickson D, Dubois B, Feldman HH, Fox NC, et al The diagnosis of mild cognitive impairment due to Alzheimer’s disease: Recommendations from the National Institute on Aging-Alzheimer’s Association workgroups on diagnostic guidelines for Alzheimer’s disease. Alzheimer’s & Dementia: the journal of the Alzheimer’s Association. 2011;7(3):270–279. 10.1016/j.jalz.2011.03.008 PMC3312027 21514249

[34] Mazzoni D. Audacity^®^; 1999-2018. https://audacityteam.org/.

[35] Roark B, Mitchell M, Hosom JP, Hollingshead K, Kaye J. Spoken language derived measures for detecting mild cognitive impairment. IEEE transactions on audio, speech, and language processing. 2011;19(7):2081–2090. 10.1109/TASL.2011.2112351 22199464 PMC3244269

[36] Gayraud F, Lee HR, Barkat-Defradas M. Syntactic and lexical context of pauses and hesitations in the discourse of Alzheimer patients and healthy elderly subjects. Clinical linguistics & phonetics. 2011;25(3):198–209. 10.3109/02699206.2010.521612 21080826

[37] Szatloczki G, Hoffmann I, Vincze V, Kalman J, Pakaski M. Speaking in Alzheimer’s disease, is that an early sign? Importance of changes in language abilities in Alzheimer’s disease. Frontiers in aging neuroscience. 2015;7:195 10.3389/fnagi.2015.00195 26539107 PMC4611852

[38] Satt A, Sorin A, Toledo-Ronen O, Barkan O, Kompatsiaris I, Kokonozi A, et al. Evaluation of speech-based protocol for detection of early-stage dementia. In: INTERSPEECH; 2013. p. 1692–1696.

[39] Boersma P. PRAAT: a system for doing phonetics by computer. Glot International. 2001;5(9/10):341–345.

[40] Meilán JJG, Martínez-Sánchez F, Carro J, López DE, Millian-Morell L, Arana JM. Speech in Alzheimer’s disease: Can temporal and acoustic parameters discriminate dementia? Dementia and Geriatric Cognitive Disorders. 2014;37(5-6):327–334. 10.1159/000356726 24481220

[41] Lopez-de Ipina K, Alonso JB, Travieso CM, Egiraun H, Ecay M, Ezeiza A, et al. Automatic analysis of emotional response based on non-linear speech modeling oriented to Alzheimer disease diagnosis. In: Intelligent Engineering Systems (INES), 2013 IEEE 17th International Conference on. IEEE; 2013. p. 61–64.

[42] De Cheveigné A, Kawahara H. YIN, a fundamental frequency estimator for speech and music. The Journal of the Acoustical Society of America. 2002;111(4):1917–1930. 10.1121/1.1458024 12002874

[43] Tsanas A, Little MA, McSharry PE, Ramig LO. Nonlinear speech analysis algorithms mapped to a standard metric achieve clinically useful quantification of average Parkinson’s disease symptom severity. Journal of the Royal Society Interface. 2011;8(59):842–855. 10.1098/rsif.2010.0456 PMC3104343 21084338

[44] Lopez-de Ipiña K, Alonso JB, Solé-Casals J, Barroso N, Henriquez P, Faundez-Zanuy M, et al On automatic diagnosis of Alzheimer’s disease based on spontaneous speech analysis and emotional temperature. Cognitive Computation. 2015;7(1):44–55. 10.1007/s12559-013-9229-9

[45] Tsanas A, Little MA, McSharry PE, Ramig LO. Accurate telemonitoring of Parkinson’s disease progression by noninvasive speech tests. IEEE Transactions on Biomedical Engineering. 2010;57(4):884–893. 10.1109/TBME.2009.2036000 19932995

[46] Khan T, Westin J, Dougherty M. Classification of speech intelligibility in Parkinson’s disease. Biocybernetics and Biomedical Engineering. 2014;34(1):35–45. 10.1016/j.bbe.2013.10.003

[47] Davis SB, Mermelstein P. Comparison of parametric representations for monosyllabic word recognition in continuously spoken sentences. In: Readings in speech recognition. Elsevier; 1990. p. 65–74.

[48] Le PN, Ambikairajah E, Epps J, Sethu V, Choi EH. Investigation of spectral centroid features for cognitive load classification. Speech Communication. 2011;53(4):540–551. 10.1016/j.specom.2011.01.005

[49] Paliwal KK. Spectral subband centroid features for speech recognition. In: Acoustics, Speech and Signal Processing, 1998. Proceedings of the 1998 IEEE International Conference on. vol. 2. IEEE; 1998. p. 617–620.

[50] Gajic B, Paliwal KK. Robust feature extraction using subband spectral centroid histograms. In: Acoustics, Speech, and Signal Processing, 2001. Proceedings.(ICASSP’01). 2001 IEEE International Conference on. vol. 1. IEEE; 2001. p. 85–88.

[51] Guyon I, Elisseeff A. An introduction to variable and feature selection. Journal of Machine Learning Research. 2003;3(Mar):1157–1182.

[52] Yu L, Liu H. Feature selection for high-dimensional data: A fast correlation-based filter solution. In: Proceedings of the 20th international conference on machine learning (ICML-03); 2003. p. 856–863.

[53] Vergara JR, Estévez PA. A review of feature selection methods based on mutual information. Neural Computing and Applications. 2014;24(1):175–186. 10.1007/s00521-013-1368-0

[54] Li T, Zhang C, Ogihara M. A comparative study of feature selection and multiclass classification methods for tissue classification based on gene expression. Bioinformatics. 2004;20(15):2429–2437. 10.1093/bioinformatics/bth267 15087314

[55] Pedregosa F, Varoquaux G, Gramfort A, Michel V, Thirion B, Grisel O, et al Scikit-learn: Machine Learning in Python. Journal of Machine Learning Research. 2011;12:2825–2830.

[56] Corp I. IBM SPSS statistics for windows, version 25.0. Armonk, NY: IBM Corp. 2017.

[57] Cawley GC, Talbot NL. On over-fitting in model selection and subsequent selection bias in performance evaluation. Journal of Machine Learning Research. 2010;11(Jul):2079–2107.

[58] Singh S, Bucks RS, Cuerden JM. Evaluation of an objective technique for analysing temporal variables in DAT spontaneous speech. Aphasiology. 2001;15(6):571–583. 10.1080/02687040143000041

[59] Fraser KC, Meltzer JA, Rudzicz F. Linguistic features identify Alzheimer’s disease in narrative speech. Journal of Alzheimer’s Disease. 2016;49(2):407–422. 10.3233/JAD-150520 26484921

[60] Tsanas A, Little MA, McSharry PE, Spielman J, Ramig LO. Novel speech signal processing algorithms for high-accuracy classification of Parkinson’s disease. IEEE transactions on biomedical engineering. 2012;59(5):1264–1271. 10.1109/TBME.2012.2183367 22249592

[61] Bennys K, Rondouin G, Vergnes C, Touchon J. Diagnostic value of quantitative EEG in Alzheimer’s disease. Neurophysiologie Clinique/Clinical Neurophysiology. 2001;31(3):153–160. 10.1016/S0987-7053(01)00254-4 11488226

[62] Brown RK, Bohnen NI, Wong KK, Minoshima S, Frey KA. Brain PET in suspected dementia: patterns of altered FDG metabolism. Radiographics. 2014;34(3):684–701. 10.1148/rg.343135065 24819789

[63] Camargo EE. Brain SPECT in neurology and psychiatry. Journal of Nuclear Medicine. 2001;42(4):611–623. 11337551

[64] Anthony JC, LeResche L, Niaz U, Von Korff MR, Folstein MF. Limits of the ‘Mini-Mental State’as a screening test for dementia and delirium among hospital patients. Psychological medicine. 1982;12(2):397–408. 10.1017/S0033291700046730 7100362

[65] Mioshi E, Dawson K, Mitchell J, Arnold R, Hodges JR. The Addenbrooke’s Cognitive Examination Revised (ACE-R): a brief cognitive test battery for dementia screening. International Journal of Geriatric Psychiatry: A journal of the psychiatry of late life and allied sciences. 2006;21(11):1078–1085. 10.1002/gps.1610 16977673

[66] Villarejo A, Puertas-Martín V. Usefulness of short tests in dementia screening. Neurología (English Edition). 2011;26(7):425–433. 10.1016/j.nrleng.2010.12.001 21345539

[67] Petersen RC, Smith GE, Waring SC, Ivnik RJ, Tangalos EG, Kokmen E. Mild cognitive impairment: clinical characterization and outcome. Archives of Neurology. 1999;56(3):303–308. 10.1001/archneur.56.3.303 10190820

[68] Faure-Bardon V, Mandelbrot L, Duro D, Dussaux C, Le M, Peytavin G. Placental transfer of elvitegravir and cobicistat in an ex-vivo human cotyledon double perfusion model. AIDS. 2018;32(3):321–325. 29112064 10.1097/QAD.0000000000001681

