# Supplementary material for: A new diagnostic approach for the identification of patients with neurodegenerative cognitive complaints
Source: PLoS One. 2019 May 24;14(5):e0217388. doi: 10.1371/journal.pone.0217388 (PMC6534304; doi:10.1371/journal.pone.0217388)
Supplement: S1 File — (PDF) [file pone.0217388.s001.pdf]

<u>Answers to the comments</u>

1) Please provide either a copy of the blank consent form provided to participants in your study or your IRB/Ethics Board approval letter.

<u>Response:</u>

We have attached a copy of participant's consent form.

2) Please provide contact information for the IRB/Ethics Board imposing ethical restrictions on the sharing of your data and confirm that they would be able to field questions from researchers who are interested in your study's data.

<u>Response:</u>

In the absence of an additional item explicitly allowing the researchers to publish the raw data or to share the raw data freely, the following sentence copied from the consent form "***I agree that the researchers can use the written, video and sound material collected in this study in anonymous form for future studies using other methods of language and communication analysis***." restricts further work on the dataset to the research team involved in the original application. It does not provide generic agreement to research by individuals not named in the ethics application.

In view of the fact that future research on the data has to include the research team involved in the initial data collection, it would be advisable for researchers interested in carrying out additional analyses on our dataset initially to contact the authors of this article. Alternatively, they could approach the ethics committee which granted approval for the original research directly. The contact details for the ethics committee are:

National Research Ethics Service (NRES) committee Yorkshire and the Humber - South Yorkshire
Millside
Mill Pond Lane
Meanwood
Leeds
LS6 4RA
