# Supplementary material for: A new diagnostic approach for the identification of patients with neurodegenerative cognitive complaints
Source: PLoS One. 2019 May 24;14(5):e0217388. doi: 10.1371/journal.pone.0217388 (PMC6534304; doi:10.1371/journal.pone.0217388)
Supplement: S2 File — (PDF) [file pone.0217388.s002.pdf]

To be printed on headed paper

CONSENT FORM

Title of the project:

Using Conversation Analysis in the differential diagnosis of memory problems: a pilot study

Please, tick initial box:

1. I confirm that I have read and understand the Information Sheet for the above study and had the opportunity to ask questions ☐

2. I understand that my participation is voluntary and that I am free to withdraw at any time, without giving any reason, without my medical care or rights being affected. ☐

3. I agree to take part in the study. ☐

4. I agree that the consultations between me and the neurologist can be audio-recorded. ☐

5. I agree that the consultation between me and the neurologist can be video-recorded. ☐

6. I agree that the researchers can use the written, video and sound material collected in this study in anonymous form for future studies using other methods of language and communication analysis. ☐

7. I agree that an anonymous form of the written, video and sound material can be used in publications about the findings of this study and to teach healthcare students and professionals. ☐

8. I agree that short, anonymous quotes from the audio or video-recorded data can be used in scientific publications arising from this study. ☐

9. I understand that relevant sections of the data collected during the study may be looked at by individuals from the University of Sheffield and the Royal Hallamshire Hospital, The University of York, and from the regulatory authorities or from the NHS Trust, where it is relevant to my taking part in this research. I give permission for these individuals to have access to my study records. ☐

10. I allow the researchers involved in this study to access my clinical records to confirm the medical diagnosis of my memory problem. ☐

Name (please print): ____

Signature: ____

Name of Person taking Consent: ____

Signature of Person taking Consent: ____
